# Dysregulated monocyte-derived macrophage response to Group B *Streptococcus* in newborns

**DOI:** 10.3389/fimmu.2023.1268804

**Published:** 2023-11-14

**Authors:** Denho Ravi, Erato Ntinopoulou, Nessim Guetta, Manuela Weier, Verena Vogel, Barbara Spellerberg, Parham Sendi, Sandrine Gremlich, Thierry Roger, Eric Giannoni

**Affiliations:** ^1^ Clinic of Neonatology, Department Mother-Woman-Child, Lausanne University Hospital and University of Lausanne, Lausanne, Switzerland; ^2^ Infectious Diseases Service, Department of Medicine, Lausanne University Hospital and University of Lausanne, Lausanne, Switzerland; ^3^ Institute of Medical Microbiology and Hygiene, University of Ulm, Ulm, Germany; ^4^ Institute for Infectious Diseases, University of Bern, Bern, Switzerland

**Keywords:** newborn, group B streptococcus, phagocytosis, cytokine, macrophage, innate immunity, streptococcus agalactiae

## Abstract

**Introduction:**

*Streptococcus agalactiae* (Group B *Streptococcus*, GBS) is a leading pathogen of neonatal sepsis. The host-pathogen interactions underlying the progression to life-threatening infection in newborns are incompletely understood. Macrophages are first line in host defenses against GBS, contributing to the initiation, amplification, and termination of immune responses. The goal of this study was to compare the response of newborn and adult monocyte-derived macrophages (MDMs) to GBS.

**Methods:**

Monocytes from umbilical cord blood of healthy term newborns and from peripheral blood of healthy adult subjects were cultured with M-CSF to induce MDMs. M-CSF-MDMs, GM-CSF- and IFNγ-activated MDMs were exposed to GBS COH1, a reference strain for neonatal sepsis.

**Results:**

GBS induced a greater release of IL-1β, IL-6, IL-10, IL-12p70 and IL-23 in newborn compared to adult MDMs, while IL-18, IL-21, IL-22, TNF, RANTES/CCL5, MCP-1/CCL2 and IL-8/CXCL8 were released at similar levels. MDM responses to GBS were strongly influenced by conditions of activation and were distinct from those to synthetic bacterial lipopeptides and lipopolysaccharides. Under similar conditions of opsonization, newborn MDMs phagocytosed and killed GBS as efficiently as adult MDMs.

**Discussion:**

Altogether, the production of excessive levels of Th1- (IL-12p70), Th17-related (IL-1β, IL-6, IL-23) and anti-inflammatory (IL-10) cytokines is consistent with a dysregulated response to GBS in newborns. The high responsiveness of newborn MDMs may play a role in the progression of GBS infection in newborns, possibly contributing to the development of life-threatening organ dysfunction.

## Introduction

Every year, 2.5 million children aged less than five years, including over half a million newborns die from sepsis (1, 2). Despite improvements in perinatal care, *Streptococcus agalactiae* (Group B *Streptococcus*, GBS) remains a leading pathogen in early-life, and a major cause of neonatal sepsis (3–5). The mortality rate of GBS neonatal sepsis is 5% in high-income countries and 20% globally, and long-term disability occurs in 20% of survivors (4, 6, 7). It is believed that the developmental status of the immune system of neonates plays a key role in the susceptibility to GBS disease (8). Yet, our limited knowledge of the reasons underlying the transition from colonization by GBS to invasion and progression to life-threatening infection hampers the development of innovative strategies targeting GBS disease.

To ensure protection against infection, newborns rely mainly on their innate immune system and bioactive molecules (such as immunoglobulins and antimicrobial peptides) transmitted from the mother through the placenta *in utero* or through breastmilk postnatally (9, 10). Quantitative and qualitative differences between the neonatal and the adult innate immune systems have been described (9, 10). Concentrations of complement proteins and other antimicrobial peptides are reduced in newborns. In addition, recruitment of neutrophils to sites of infection and ability to phagocytose and kill pathogens are limited. Newborn dendritic cells and monocytes exposed to lipopolysaccharides (LPS) release lower amounts of the pro-inflammatory and Th1-polarizing cytokines tumor necrosis factor (TNF), IL-1β, IL-12p70, but similar or even higher levels of Th17-polarizing and anti-inflammatory cytokines IL-6, IL-23 and IL-10 compared to adult cells (11–16). However, the neonatal immune responses to GBS might differ from responses to purified microbial products (17–20). Moreover, newborns affected by GBS disease have strong systemic pro- and anti-inflammatory responses (21, 22), consistent with the observation that a dysregulated host response to infection contributes to the pathogenesis of sepsis (23).

Macrophages are distributed across all tissues, serving as resident innate immune cells (24). The differentiation, activation and function of macrophages are shaped by environmental cues, spanning from the induction of classically activated pro-inflammatory M1 macrophages to the promotion of alternatively activated pro-resolving/anti-inflammatory M2 macrophages (25). Macrophages play a crucial role to orchestrate host immunity and inflammatory response with the release of a large panel of cytokines (24). In murine models of pneumonia, newborn alveolar macrophages have a lower capacity to phagocytose and kill GBS compared to adult cells, suggesting that reduced macrophage responses might contribute to the vulnerability of newborns to this pathogen (26, 27). Yet, studies investigating the responses of human newborn macrophages to GBS are lacking.

Given the importance of macrophages in host defenses against bacteria and in the pathogenesis of sepsis (23), a deeper understanding of the interactions between GBS and newborn macrophages is needed. We developed a robust *in vitro* model to compare the responses of primary human newborn and adult monocyte-derived macrophages (MDMs) exposed to GBS. The phagocytosis, bacterial killing, production of cytokines and viability of MDMs was quantified under different conditions of macrophage activation. Here, we report a picture consistent with a dysregulated cytokine response with a conserved capacity to phagocytose and kill GBS in newborns.

## Materials and methods

### Ethical statement

The study protocol was approved by the ethics committee of the Canton de Vaud, Switzerland (CER-VD, project #2019-01772). Written informed consent was obtained from the mothers for the collection of umbilical cord blood and from adult volunteers for peripheral blood.

### Blood samples

Umbilical cord blood was collected from 20 healthy term newborns by puncture of placental vessels, after delivery of the placenta. Blood was obtained from 18 healthy adult volunteers (age 20-59 years) by puncture of a peripheral vein. S-Monovettes^®^ containing EDTA (Sarstedt AG & Co. KG) were used to collect blood.

### Isolation, culture and activation of cells

Mononuclear cells were isolated from blood by gradient density centrifugation using Ficoll-Paque™ PLUS solution (GE Healthcare). CD14+ monocytes were purified from mononuclear cells by positive selection using magnetic microbeads coupled to anti-human CD14 antibodies (Miltenyi Biotec) (28–30). Cells were incubated with Pacific Blue™ anti-human CD14 antibody (BioLegend) to determine the purity of each monocyte preparation. Data were acquired using an Attune Nxt Flow Cytometer (ThermoFisher) and analysed using FlowJo (version 10.7, FlowJo LLC). For experiments, we only used monocyte preparations with a purity above 90%. The viability of mononuclear cells and monocytes was determined by trypan blue exclusion using a CountessTM Automated cell counter (Invitrogen). Monocytes (10^5^ cells/well) were cultured in 96 well plates for seven days at 37°C, 5% CO_2_, in RPMI 1640 (ThermoFisher) supplemented with 10% native human serum (HS, from human male AB plasma, Sigma-Aldrich Corp.), 1% penicillin-streptomycin (ThermoFisher) and 50 ng/ml human macrophage colony-stimulating factor (M-CSF, PeproTech EC Ltd.) to induce their differentiation into monocyte-derived macrophages (MDMs). After 7 days, the medium was changed to RPMI containing 10% HS and different concentrations ranging from 10 to 100 ng/ml (50 ng/ml if not otherwise stated) of human M-CSF, granulocyte-macrophage colony-stimulating factor (GM-CSF, PeproTech EC Ltd.) or interferon gamma (IFNγ, PBL Assay Science) (30). Cells were incubated for 24 hours to induce GM-CSF- or IFNγ-activated MDMs (GM-CSF-MDMs, IFNγ-MDMs), or resting macrophages (M-CSF-MDMs) (31).

### Preparation of GBS COH1

All experiments were performed with GBS COH1 (American Type Culture Collection BAA-1176), a serotype III sequence type 17 reference strain obtained from a newborn with sepsis (32). We incubated bacteria overnight at 37°C, with 5% CO_2_ in Brain Heart Infusion (BHI, ThermoFisher) broth. New tubes containing BHI medium were inoculated with overnight cultures (1:40) and incubated for two hours, to reach mid-log phase of growth, corresponding to 6 x 10^8^ bacteria/ml. Bacteria were washed and resuspended in ice-cold phosphate-buffered saline solution (PBS) at 2 x 10^5^ - 10^7^ bacteria/100 µl. GBS was plated on Columbia III agar with 5% sheep blood (BD Biosciences) to determine the concentration of our bacterial culture. The minimum inhibitory concentration (MIC) of GBS COH1 to gentamicin was 64 µg/ml. MIC was determined by overnight BHI liquid cultures of GBS COH1 in 96 well plates (37°C, 5% CO_2_) with serial dilution of gentamicin (Sigma-Aldrich) ranging from 0.5 to 512 µg/ml. At the end of incubation, proliferation of bacteria was determined by optical density (O.D. 600 nm).

### Cytokine measurements

M-CSF-, GM-CSF- and IFNγ-MDMs were exposed to 10^7^ GBS per well or 0.2 µg/ml lipopolysaccharide (LPS, Invivogen), or 2 µg/ml Pam3CysSerLys4 (Pam_3_CSK_4_, Invivogen). After one hour of incubation, we added gentamicin to reach a final concentration of 100 µg/ml. Cell-culture supernatants were collected after 18 hours to quantify cytokines by ELISA (BD Biosciences, for human TNF) and by ProcartaPlex immunoassays (Human Custom ProcartaPlex 16-plex, Invitrogen, for IL-1β, IL-1RA, IL-6, IL-8, IL-10, IL-12p70, IL-18, IL-20, IL-21, IL-22, IL-23, IL-27, MCP-1, MIF, RANTES, and IFNβ).

### Gentamicin protection assay

The principle behind this method is to count bacteria (colony-forming units: CFU) inside macrophages after precise intervals of time in order to determine phagocytosis and intracellular killing of bacteria (33). Gentamicin is used to kill bacteria that are outside but not those that are inside macrophages, preventing extracellular growth of bacteria. M-CSF-, GM-CSF-, and IFNγ-MDMs in 96 well plates were exposed to 2 x 10^5^ GBS/well to determine phagocytosis and intracellular killing. Plates were centrifuged 15 minutes at 50 RCF and 37°C to maximize contact between bacteria and MDMs, and incubated for 45 minutes. Cells were washed twice with warm PBS, and incubated 30 minutes in RPMI 10% HS containing 10-100 ng/ml M-CSF, GM-CSF or IFNγ and 100 µg/ml gentamicin to kill the remaining extracellular bacteria (33). Phagocytosis was determined after a washing step and lysis of MDMs with sterile water followed by plating of serial dilutions of cell lysates on Columbia III agar with 5% sheep blood plates ( $\text{\% phagocytosis}=\;\frac{\text{mean of counted CFU}}{\text{initial inoculum}}$ ). A second and a third plate were incubated with RPMI 10% HS, 20 µg/ml gentamicin and 10-100 ng/ml M-CSF, GM-CSF or IFN-γ for 3 and 18 hours. Intracellular bacteria were quantified as described above, and data was presented relative to the number of phagocytosed bacteria ( $\text{\% survival}=\;\frac{\text{mean of CFU in phagocytosis}}{\text{mean of CFU in survival}}$ ).

To validate the model, we quantified by plating the extracellular bacteria remaining after antibiotic treatment and washing of cell cultures. We also quantified the intracellular concentration of gentamicin by fluorescence polarization immunoassay (FPIA) using a Cobas Integra 400 plus (Roche). In additional validation steps, we inhibited phagocytosis by replacing HS with heat-inactivated fetal bovine serum (FBS, HyClone ThermoFisher), and by treating MDMs with 1, 5 and 10 µM cytochalasin D (*Zigosporium mansonii*, Merck & Cie), 1 and 10 µM oligomycin (*Streptomyces diastatochromogenes*, Sigma-Aldrich), and 10 and 50 mM 2-deoxy-D-glucose (2-DG, Roth AG) alone or in combination for 30 minutes (cytochalasin D and FBS) or one hour (oligomycin and 2-DG) before exposition to GBS.

### Fluorescence microscopy

GBS was stained by incubating bacteria for 1 hour at 25°C with fluorescein isothiocyanate (FITC), followed by four washing steps (34). MDMs were exposed to FITC-labeled GBS and pictures of cells were taken at 1 hour with an EVOS M7000 Imaging System (ThermoFisher). Phagocytosis of FITC-labeled GBS was quantified in 100 macrophages. The viability of MDMs was assessed in cytokine (MOI 100) and inhibitor (MOI 2) plates, using the LIVE/DEAD™ Cell Imaging kit (Life-Technologies). The number of live and dead MDMs was quantified based on analysis of the pictures acquired with the EVOS M7000 Imaging System.

### Statistical analysis

Analyses and graphs were performed using GraphPad Prism (GraphPad Software 9.5.1) and Microsoft Excel software. Data are expressed as means ± SEMs. Groups were compared by two-way ANOVA followed by Dunnett’s and Sidak’s multiple comparison tests, to compare each experimental condition with the control condition within the same donor group and to compare the newborns to the adults for a same experimental condition. Results were considered statistically significant when P < 0.05.

## Results

### Activation of newborn and adult MDMs by IFNγ increases GBS-induced TNF production

**Figure 1:**
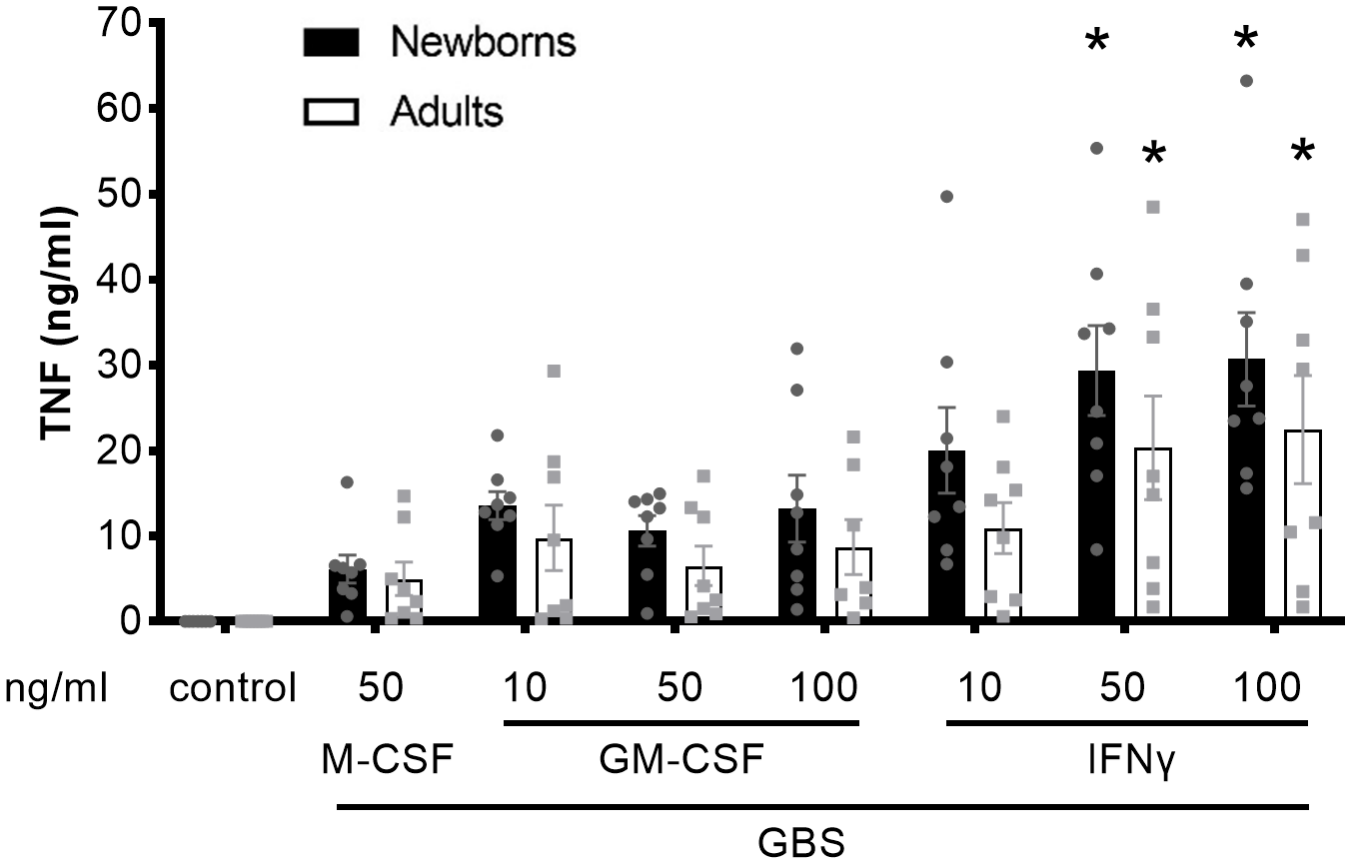
Activation of newborn and adult MDMs by IFNγ increases GBS-induced TNF production. Newborn and adult resting (M-CSF-) and GM-CSF- or IFNγ-activated MDMs were exposed to GBS (10^7^ bacteria, MOI 100). The control condition corresponds to 50 ng/ml M-CSF-MDMs not exposed to GBS. Concentrations of TNF were measured in cell culture supernatants collected at 18 hours. Results are expressed as mean ± SEM of 8 newborns and 8 adults. Analysis by two-way ANOVA followed by Dunnett’s multiple comparison test to assess differences with the control condition of the same age group is presented. *P < 0.05 vs M-CSF-MDMs exposed to GBS.

We compared the capacity of resting M-CSF-MDMs, GM-CSF-activated, and IFN-γ-activated newborn and adult MDMs to release TNF in response to live GBS COH1 (**Figure 1**). In comparison with resting M-CSF-MDMs, IFNγ-activated newborn and adult MDMs secreted 3.3- and 4.5-fold higher amounts of TNF in response to GBS. The trends towards more TNF production by GM-CSF-activated compared to resting MDMs and towards a greater production of TNF by newborn compared to adult MDMs did not reach statistical significance.

### Newborn MDMs release higher level of IL-1β, IL-6, IL-10, IL-12p70 and IL-23 than adult MDMs in response to GBS

**Figure 2:**
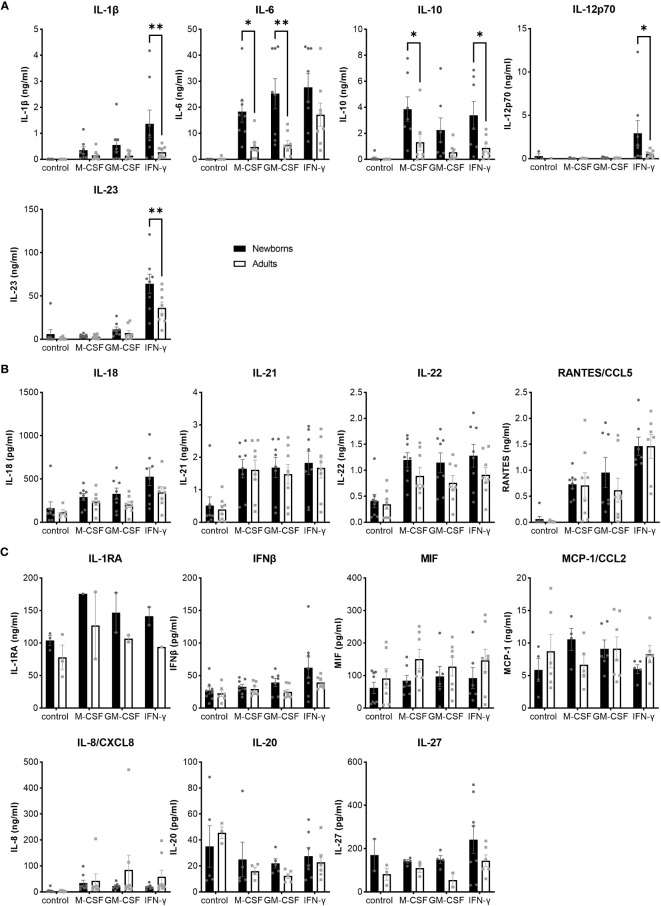
Distinct pattens of cytokine and chemokine release by newborn and adult MDMs in response to GBS. Newborn and adult resting (M-CSF-) and GM-CSF- or IFNγ-activated MDMs were exposed to GBS (10^7^ bacteria, MOI 100). The control condition corresponds to M-CSF-MDMs not exposed to GBS. Concentrations of M-CSF, GM-CSF and IFNγ were 50 ng/ml. Concentrations of 16 cytokines/chemokines were measured in cell culture supernatants collected at 18 hours. **(A)** Cytokines differentially induced in newborn and adult MDMs: IL-1β, IL-6, IL-10, IL-12p70, IL-23. **(B)** Cytokines/chemokines induced at similar levels in newborn and adult MDMs: IL-18, IL-21, IL-22, RANTES/CCL5. **(C)** Cytokines/chemokines not induced by GBS, neither in newborn nor in adult MDMs. Results are expressed as mean ± SEM of 8 newborns and 8 adults (except for IL-1RA in panel C, n = 2-3). Analysis by two-way ANOVA followed by Sidak’s multiple comparison test to assess differences for the same condition between newborns and adults is presented. *P < 0.05, **P < 0.01.

**Figure 3:**
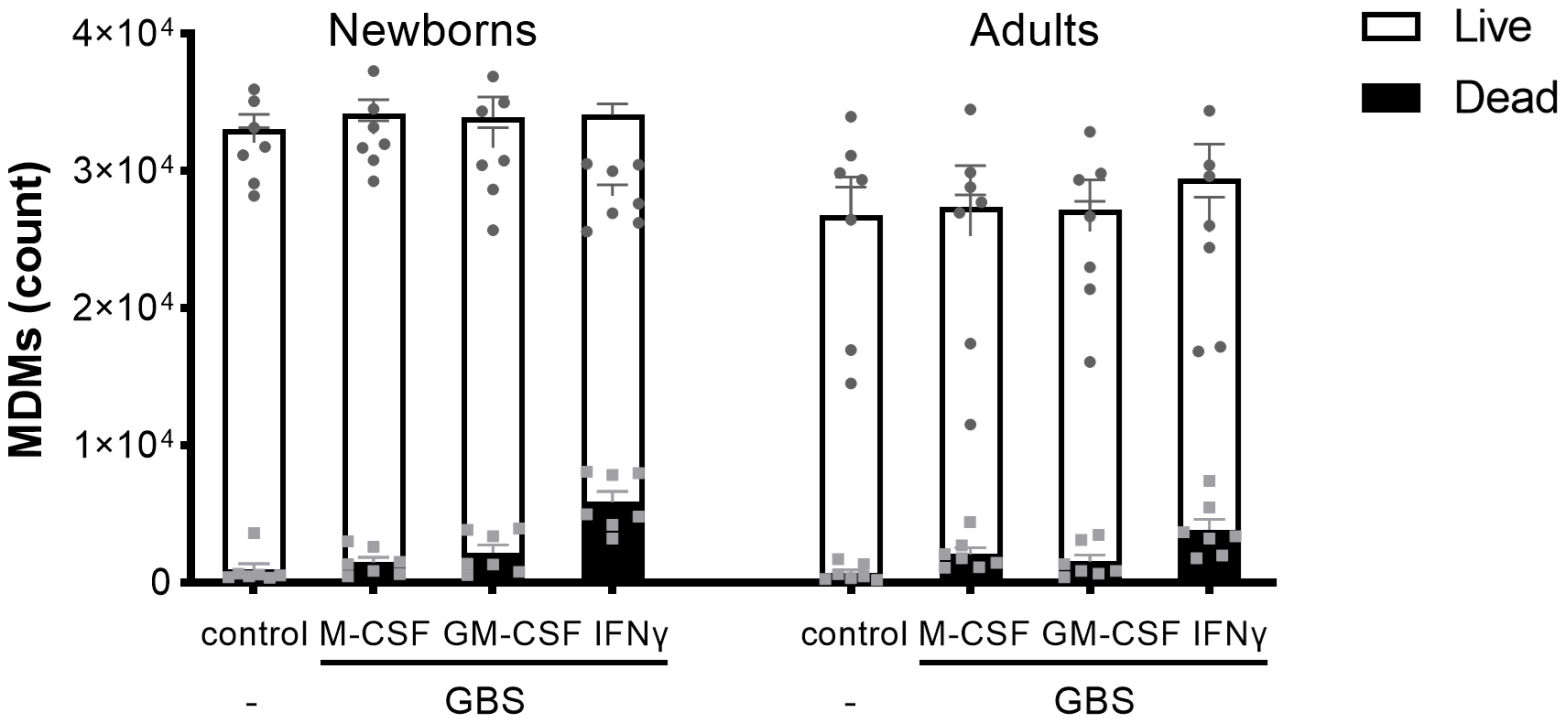
Newborn and adult MDMs maintain the same viability after exposure to GBS. Newborn and adult resting (M-CSF-) and GM-CSF- or IFNγ-activated MDMs were exposed to 10^7^ GBS (MOI 100). The control condition corresponds to M-CSF-MDMs not exposed to GBS. Concentrations of M-CSF, GM-CSF and IFNγ were 50 ng/ml. The number of live and dead newborn and adult MDMs was quantified by fluorescence microscopy after 18 hours of incubation with GBS. Results are expressed as mean ± SEM of 7 newborns and 7 adults. Analysis by two-way ANOVA followed by Sidak’s multiple comparison test to assess differences for the same condition between newborns and adults is presented. *P < 0.05.

To have a broad view on host responses, we quantified by multiplex immunoassay the secretion of 16 cytokines and chemokines by newborn and adult M-CSF-, GM-CSF- and IFNγ-MDMs exposed to live GBS COH1 (**Figure 2**). We presented the cytokines induced by GBS with a difference between newborn and adult MDMs in **Figure 2A**, the cytokines induced by GBS with no difference between newborn and adult MDMs in **Figure 2B**, and the cytokines not induced by GBS in **Figure 2C**. Exposure to GBS led to the secretion of IL-1β, IL-6, IL-10, IL-12p70, IL- 18, IL-21, IL-22, IL-23, and RANTES/CCL5, but not IL-1RA, IL-8/CXCL8, IL-20, IL-27, IFN-β, MCP-1/CCL2 and MIF in newborn and adult MDMs. GBS-induced secretion of IL-1β, IL-12p70 and IL-23 was more elevated in IFNγ- than in M-CSF-MDMs. Compared to adults, newborn M-CSF-MDMs exposed to GBS produced higher levels of IL-6 (3.8-fold) and IL-10 (3-fold), newborn GM-CSF-MDMs produced higher levels of IL-6 (4.5-fold), and newborn IFNγ-MDMs produced higher levels of IL-1β (4.9-fold), IL-12p70 (5.7-fold), IL-10 (3.8-fold) and IL-23 (1.8-fold). To evaluate the specificity of host responses to GBS, we exposed IFNγ-MDMs to the Toll-like receptor (TLR)-4 ligand LPS and the TLR1/2 ligand Pam_3_CSK_4_ (**Supplementary Figure 1**). LPS induced a higher production of IFNβ (2.5-fold) and IL-10 (10.6-fold) in newborn compared to adult IFNγ-MDMs. No difference in the secretion of the 16 cytokines and chemokines was observed between newborn and adult IFNγ-MDMs exposed to Pam_3_CSK_4_. We quantified the number of live and dead cells 18 hours after exposure to GBS to investigate a potential impact of cell survival on the differential responses of newborn and adult MDMs. Viability at 18 hours was 95 ± 2% and 92 ± 4% for newborn and adult M-CSF-MDMs, 94 ± 5% and 93 ± 6% for newborn and adult GM-CSF-MDMs, and 84 ± 5% and 87 ± 8% for newborn and adult IFNγ-MDMs (**Figure 3**). Normalizing cytokine levels according to the number of cells in each well (**Supplementary Figure 2**) did not modify our findings.

### Newborn and adult MDMs have a similar rate of phagocytosis and killing of GBS

**Figure 4:**
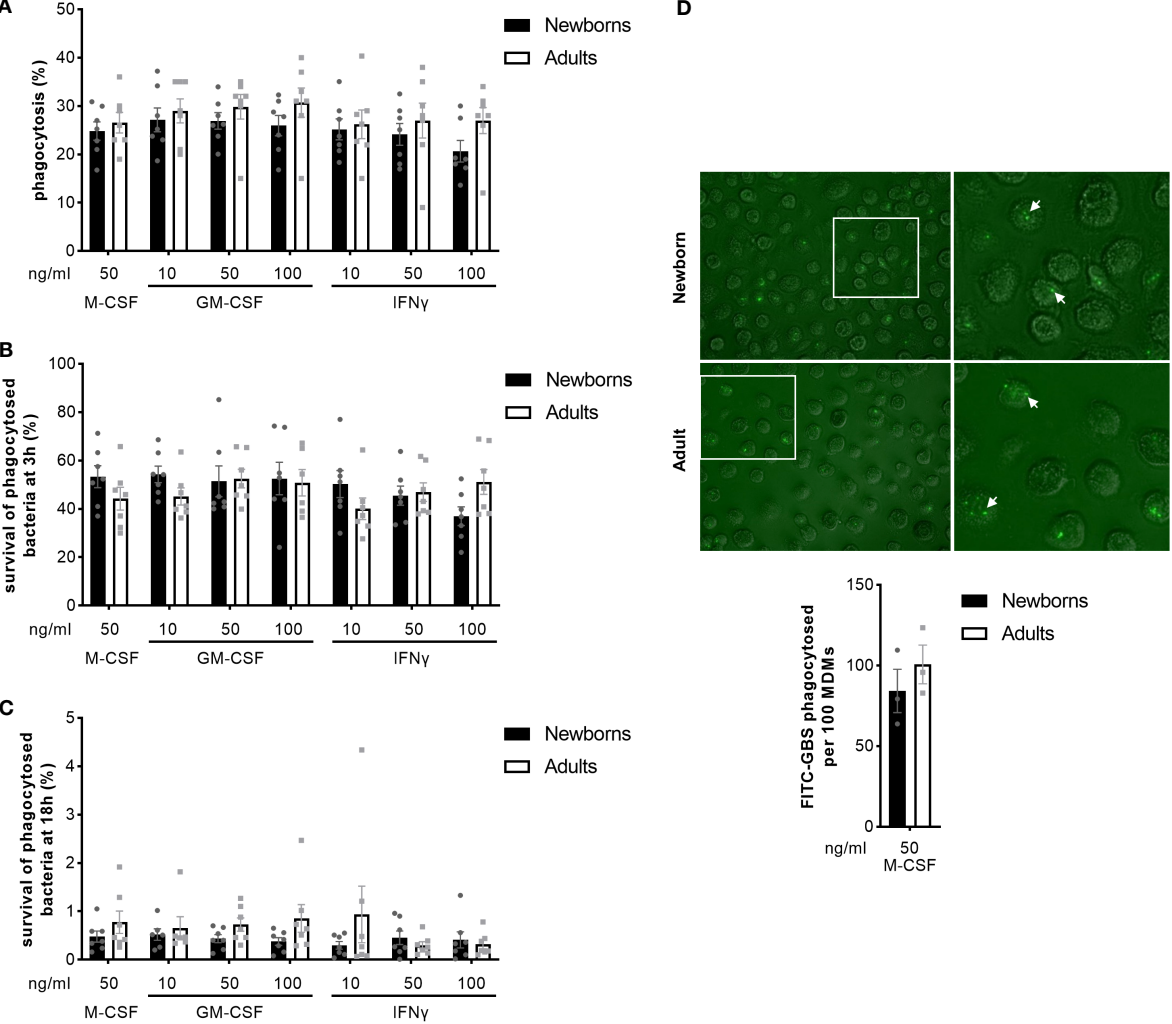
Newborn and adult MDMs phagocytose and kill GBS at a similar rate. Newborn and adult resting (M-CSF-) and GM-CSF- or IFNγ-activated MDMs were exposed to 2 x 10^5^ GBS (MOI 2) during 1 hour. Phagocytosis of GBS was quantified by plating cell lysates **(A)**. Intracellular survival of GBS was quantified by plating cell lysates at 3 **(B)** and 18 hours **(C)**. Phagocytosis of GBS was visualized by fluorescence microscopy. Representative images and quantification of FITC-labelled GBS internalized by newborn and adult M-CSF-MDMs are shown, with a 100x magnification. The white arrows indicate phagocytosed bacteria. **(D)**. Results are expressed as mean ± SEM of 7 newborns and 7 adults **(A–C)** and 3 newborns and 3 adults **(D)**. Analysis by two-way ANOVA followed by Sidak’s multiple comparison test to assess differences for the same condition between newborns and adults is presented. *P < 0.05.

We analyzed the capacity of newborn and adult MDMs to phagocytose and kill GBS COH1 using a gentamicin protection assay. Newborn and adult M-CSF-MDMs phagocytosed 25 ± 5% and 27 ± 6% of the inoculum (**Figure 4A**). At 3 hours, 53 ± 12% and 44 ± 12% of phagocytosed bacteria survived in newborn and adult M-CSF-MDMs (**Figure 4B**). At 18 hours, survival of bacteria was drastically reduced to < 2% (**Figure 4C**), showing that both newborn and adult MDMs efficiently killed internalized bacteria. Activation of MDMs by GM-CSF or IFNγ at concentrations between 10 and 100 ng/ml did not influence phagocytosis or killing of GBS. We confirmed the absence of difference in phagocytosis between newborn and adult M-CSF-MDMs by quantifying 84 ± 13 and 100 ± 12 FITC-labeled GBS phagocytosed per 100 newborn and adult MDMs (**Figure 4D**).

To validate our observations, we addressed potential methodological drawbacks: non-specific killing of intracellular bacteria by internalized gentamicin, and non-exhaustive killing of extracellular and membrane-bound bacteria. Intracellular gentamicin concentrations measured in newborn and adult M-CSF-MDMs at the end of the killing assay were below 0.1 µg/ml (**Supplementary Table I**), in line with the notion that gentamicin has a slow rate of entry into eukaryotic cells (35). Since we measured the minimal inhibitory concentration (MIC) of gentamicin for GBS COH1 to be 64 µg/ml, intracellular bacteria could not be affected by gentamicin. Exhaustive killing of extracellular and membrane-bound bacteria was verified by plating the cell culture supernatant 3 and 18 hours after gentamicin treatment. For newborn and adult MDMs, the proportion of live extracellular bacteria was < 0.5% of the inoculum used for infection (**Supplementary Table II**).

### Opsonization enhances while inhibition of actin polymerization or energy metabolism prevents phagocytosis of GBS

**Figure 5:**
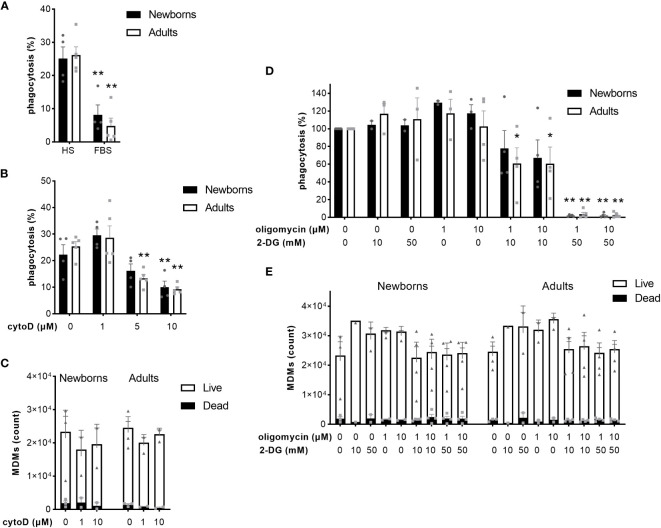
Opsonization, actin polymerization and energy metabolism contribute to phagocytosis of GBS by newborn and adult MDMs. Newborn and adult M-CSF-MDMs were pre-incubated with 10% native human serum (HS) **(A-E)** or 10% heat-inactivated fetal bovine serum (FBS) **(A)**, with increasing concentrations of cytochalasin D (cytoD) for 30 minutes **(B, C)**, oligomycin **(D, E)** and 2 deoxy-glucose (2-DG) **(D, E)** for one hour, or vehicle (DMSO for oligomycin or cytoD) and then exposed to 2 x 10^5^ GBS (MOI 2) during 1 hour. Phagocytosis of GBS was quantified by plating cell lysates **(A, B, D)**. The number of live and dead MDMs was quantified by fluorescence microscopy **(C, E)**. Results are expressed as mean ± SEM **(A–C, E)** or normalized to controls **(D)** of 5 newborns and 5 adults. Analysis by two-way ANOVA followed by Dunnett’s multiple comparison test to assess differences with the control condition within the same age group is presented. *P < 0.05, **P < 0.01 vs controls.

The coating of pathogens with opsonins, followed by reorganization of the actin cytoskeleton, and metabolic reprogramming of macrophages are key steps to ensure optimal phagocytosis of bacteria (36). Given that previous research has focused on murine macrophages and cell lines, we investigated the impact of opsonization, actin polymerization, glycolysis and oxidative phosphorylation on the capacity of newborn and adult MDMs to phagocytose GBS. We quantified GBS uptake in the presence of heat-inactivated FBS, which contains very low concentrations of opsonins (37). Replacement of native HS by heat-inactivated FBS decreased GBS phagocytosis from 25 ± 7% to 8 ± 6% in newborn and from 26 ± 6% to 5 ± 5% in adult M-CSF-MDMs (**Figure 5A**). Pre-incubation of M-CSF-MDMs with 1, 5 and 10 µM cytochalasin D, an inhibitor of actin polymerization, hindered phagocytosis in a dose-dependent manner. Cytochalasin D at 10 µM reduced phagocytosis by newborn and adult MDMs from 26 ± 6% to 10 ± 5% and from 28 ± 6% to 9 ± 2% (**Figure 5B**), without significantly affecting cell viability (**Figure 5C**). Inhibition of glycolysis with 2-DG or inhibition of oxidative phosphorylation by oligomycin did not affect GBS phagocytosis (**Figure 5D**). However, the combination of both inhibitors elicited a large and dose-dependent reduction of phagocytosis (**Figure 5D**), with a 39% ± 18% and 39% ± 19% decrease in phagocytosis at 10 mM 2-DG and 1-10 µM oligomycin in adult MDMs, and a 98 ± 0.5% and 98% ± 1%, 96% ± 2% and 97% ± 1% decrease at 50 mM 2-DG and 1-10 µM oligomycin for newborn and adult M-CSF-MDMs. Both inhibitors, either alone or in combinations, at concentrations up to 50 mM for 2-DG and 10 µM for oligomycin had no impact on cell viability (**Figure 5E**).

## Discussion

GBS causes a major burden of disease in early-life, including stillbirths, neonatal and infant mortality, and long-term disability (3–6). Here, we show that GBS induces a distinct host response in newborn and adult MDMs, with differences depending on the conditions of macrophage activation. Overall, newborn MDMs exposed to GBS release higher amounts of Th1, Th17 and anti-inflammatory cytokines and phagocytose and kill the bacteria to the same extent as adult MDMs.

Depending on the state of macrophage activation, live GBS triggers a greater release of IL-1β, IL-6, IL-10, IL-12p70, and IL-23 in newborn compared to adult MDMs. IL-12p70 is necessary to drive the differentiation of Th1 cells, while IL-1β, IL-6 and IL-23 are required to induce and maintain Th17 cell differentiation (38). IL-10 dampens inflammatory responses, thereby limiting tissue damage. Therefore, our results point towards greater Th1, Th17, and anti-inflammatory responses in newborn MDMs. These findings contrast with previous studies indicating that neonatal monocytes and dendritic cells exposed to TLR 1/2, 3, 4, 7/8, 8 and 9 agonists release lower amounts pro-inflammatory and Th1-polarizing cytokines than adult cells (11–16). The most consistent observation reported in the literature is the reduced capacity of neonatal innate immune cells to release IL-12p70 in response to TLR agonists (12, 15, 16, 39). Innate immune cells detect live bacteria through multiple pattern recognition receptors. Studies conducted in animal models and immortalized cell lines indicate that innate immune cells sense GBS through TLR2/6, TLR7, TLR8, TLR9, cyclic GMP-AMP synthase (cGAS) and NOD-like receptor family, pyrin domain containing 3 (NLRP3) (40–46). Mixed populations of newborn mononuclear cells (MNCs) produce lower levels of IL-10, IL-18 and IFNγ but similar levels of TNF and IL-6 compared to adult cells in response to heat killed GBS (47–50). Our results clearly show that cytokine responses of MDMs to live GBS are different from those to purified TLR2 or TLR4 agonists or heat inactivated bacteria, emphasizing the importance of using live bacteria in experimental models of infection.

Murine newborn macrophages have reduced capacities to phagocytose and kill bacteria, including GBS (26, 27, 51, 52). Conversely, human newborn and adult MNCs (19, 53) and MDMs (our data) internalize and eliminate GBS to the same extent. Opsonization enhances phagocytosis of GBS by murine macrophages and human MNCs from adults (54, 55). Our results suggest that opsonization may play an important role in promoting the uptake of GBS by primary human MDMs. In addition, newborn MDMs exhibit comparable efficiency as adult MDMs in internalizing GBS across various opsonization conditions. This suggests that newborn MDMs do not have an intrinsically reduced capacity to phagocytose GBS. Phagocytosis is a high energy-requiring process. During bacterial infection, a metabolic shift reprograms macrophages towards aerobic glycolysis (56). While high glycolytic activity is associated with efficient phagocytosis in murine macrophages (57–59), the impact of oxidative phosphorylation on phagocytosis has not been investigated. We report that inhibitors of both glycolysis and oxidative phosphorylation are required to reduce phagocytosis by newborn and adult MDMs, in line with the importance of metabolic pathways to supply the energy required for the uptake of bacteria. Previous studies have shown that GBS can survive in macrophage cell lines (60–62) and can induce macrophage cell death (63, 64). Our data indicate that primary human MDMs from newborns and adults are able to fully eliminate phagocytosed GBS without an impact on the viability of MDMs. Therefore, the vulnerability of neonates to GBS disease may not be related to a reduced capacity of macrophages to phagocytose and kill the bacteria.

During infection, Th1, Th17 and anti-inflammatory responses are required to initiate, amplify, and terminate the responses required to clear pathogens while minimizing tissue damage. Th1 CD4+ T cells provide protection against intracellular pathogens through the production of IFNγ and IL-2. IFNγ promotes bacterial clearance of GBS in newborn mice by increasing the bactericidal capacity of whole blood and peritoneal macrophages (62, 65). Given that activation by IFNγ does not increase the bactericidal capacity of human MDMs, the strong production of Th1 cytokines by newborn MDMs *in vitro* may not translate into enhanced protective responses during GBS infection. Th17 CD4+ T cells confer protection against bacteria through early recruitment of neutrophils and other inflammatory cells (66). However, naïve human neonatal CD4+ T cells exposed to IL-1β, IL-6 and IL-23 preferentially adopt an immunoregulatory Th22 phenotype, rather than a stereotypic Th17 phenotype (67). The higher production of Th1, Th17 and anti-inflammatory cytokines in newborns compared to adult MDMs in response to GBS challenges the concept that the neonatal immune system is poorly responsive, in line with recent literature (29, 68, 69). This may represent an age-adapted protective response to a dangerous pathogen. Alternatively, our findings are consistent with a dysregulated neonatal immune response to GBS. Indeed, a dysregulated host response, characterized by excessive activation of the immune system, concomitantly with features of immune suppression, plays a central role in the pathogenesis of sepsis, and sepsis-related adverse outcomes in adults (23). In accordance with this concept, GBS infection is associated with strong systemic pro- and anti-inflammatory responses in newborns (21, 22, 70).

Previous studies in humans have investigated mixed populations of umbilical cord blood MNCs or monocyte-derived cells, without a phenotypic characterization of differentiated cells or validation of phagocytosis and killing assays. Several validation steps ensured the robustness of our model. The viability of MDMs obtained from highly purified preparations of CD14+ monocytes was confirmed in each experiment. The production of a large number of cytokines by MDMs was quantified in several conditions of activation. We addressed the potential pitfalls of the gentamicin protection assay by multiple validation experiments.

Our study has several limitations. An *in vitro* system does not reflect the tissue-specific environment of macrophages *in vivo*. For example, newborns have low circulating levels of opsonins (71). In our experiments, the use of human adult serum or heat inactivated FBS in the culture medium provided the same amount of opsonins for newborn and adult MDMs, allowing to analyze their intrinsic phagocytic capacities. However, we did not test the impact of specific opsonins in the phagocytosis of GBS. Although birth is the time of life when the risk of developing GBS infection is highest, studying children could inform on changes reflecting the transition from a newborn GBS-sensitive to an adult GBS-resistant immune system. While GBS COH1 is a reference strain for neonatal studies, it may not be representative of the strains that are causing GBS infection nowadays. Moreover, we did not assess the mechanisms underlying the differences in cytokine production observed between newborn and adult MDMs.

In summary, we provide an extensive description of a robust methodology aimed at evaluating the cytokine response and the capacity of newborn and adult MDMs to phagocytose and kill GBS. We show that newborn MDMs have an increased Th1- and Th17-related cytokine response, together with enhanced release of IL-10 in response to GBS. Newborn MDMs do not have an intrinsically reduced capacity to phagocytose or kill GBS. These findings indicate that the neonatal innate immune system is highly responsive to GBS, and point towards a dysregulated macrophage response to this bacterium in newborns. This may have strong implications for the understanding of the mechanisms underlying the progression towards life-threatening organ dysfunction during GBS infection. Additionally, our results could be relevant for the development of immune modulating therapies.

## Data availability statement

The original contributions presented in the study are included in the article/**Supplementary Material**, further inquiries can be directed to the corresponding author/s.

## Ethics statement

The studies involving humans were approved by Ethics Committee on human research of the Canton de Vaud (CER-VD). The studies were conducted in accordance with the local legislation and institutional requirements. Written informed consent for participation in this study was provided by the participants’ legal guardians/next of kin.

## Author contributions

DR: Writing – original draft, Data curation, Methodology, Writing – review & editing, Conceptualization, Formal Analysis, Investigation. EN: Writing – review & editing, Formal Analysis, Investigation. NG: Formal Analysis, Investigation, Writing – review & editing. MW: Investigation, Writing – review & editing. VV: Writing – review & editing, Formal Analysis. BS: Formal Analysis, Writing – review & editing, Conceptualization, Methodology. PS: Conceptualization, Formal Analysis, Methodology, Writing – review & editing. SG: Formal Analysis, Methodology, Writing – review & editing, Investigation, Supervision, Writing – original draft. TR: Conceptualization, Formal Analysis, Methodology, Supervision, Writing – review & editing. EG: Conceptualization, Formal Analysis, Funding acquisition, Investigation, Methodology, Resources, Supervision, Writing – review & editing.
