# Supplementary figures and images for: Dysregulated monocyte-derived macrophage response to Group B Streptococcus in newborns
Source: Front Immunol. 2023 Nov 14;14:1268804. doi: 10.3389/fimmu.2023.1268804 (PMC10682703; doi:10.3389/fimmu.2023.1268804)

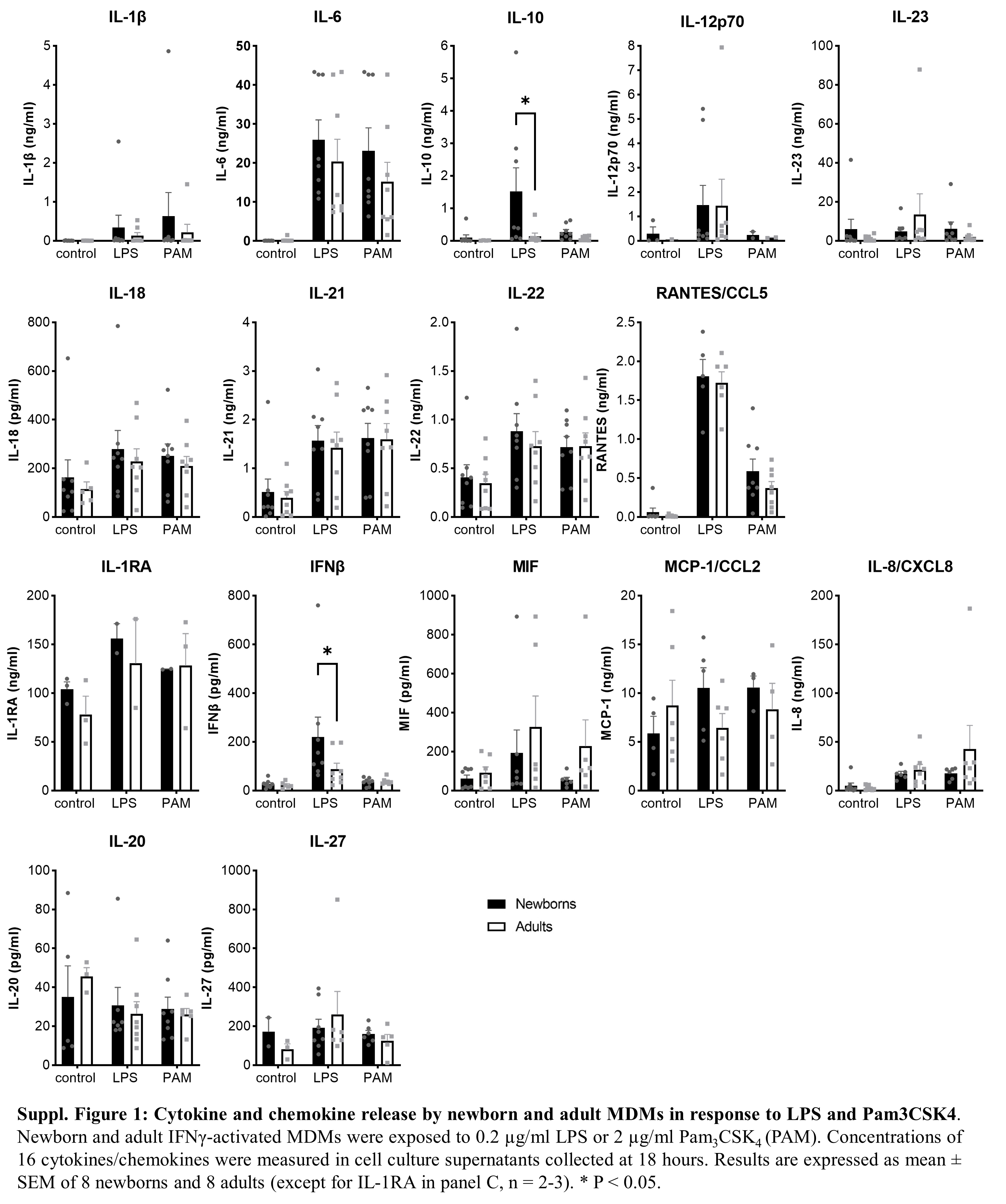

Supplement: Supplementary file 1 [file Image_1.tif]

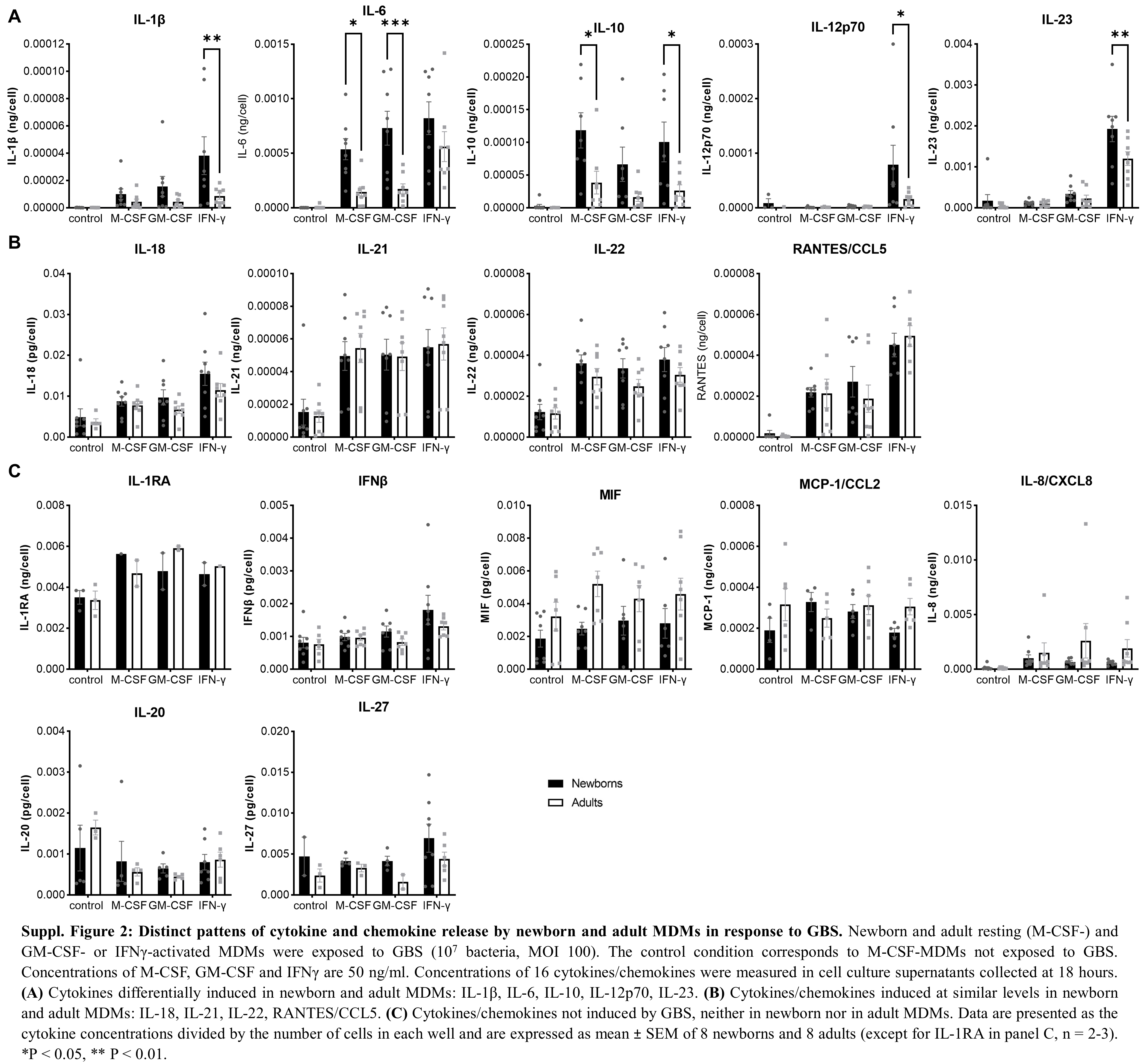

Supplement: Supplementary file 2 [file Image_2.tif]
