# Supplementary material for: Dysregulated monocyte-derived macrophage response to Group B Streptococcus in newborns
Source: Front Immunol. 2023 Nov 14;14:1268804. doi: 10.3389/fimmu.2023.1268804 (PMC10682703; doi:10.3389/fimmu.2023.1268804)
Supplement: Supplementary file 3 [file Table_1.docx]

**Supplementary Table I:** Intracellular gentamicin concentration was quantified in newborn and adult M-CSF-MDMs after 30 minutes incubation with 100 µg/ml gentamicin, and further incubation with 20 µg/ml gentamicin to reach 3 or 18 hours. Results are expressed as mean of triplicates of 2 newborns and 2 adults.

| **Gentamicin (µg/ml)** | **Newborns** | **Adults** |
| --- | --- | --- |
| **3h** | < 0,04 / 0,08 | < 0,04 / < 0,04 |
| **18h** | < 0,04 / 0,08 | < 0,04 / < 0,04 |

**Supplementary Table II**: Extracellular bacteria (percentage of the initial inoculum) were quantified after 1h of exposition of newborn and adult M-CSF-MDMs to GBS, 30 minutes incubation with 100 µg/ml gentamicin, and further incubation with 20 µg/ml gentamicin to reach 3 or 18 hours. Results are expressed as mean ± SEM of triplicates of 6 newborns and 6 adults for the 3 hours, and 2 newborns and 2 adults for the 18 hours time points.

| **% of extracellular GBS** | **Newborns** | **Adults** |
| --- | --- | --- |
| **3h** | 0.22 ± 0,13 % | 0,31 ± 0,21 % |
| **18h** | 0.002 ± 0,001 % | 0.001 ± 0,0002 % |
